# Using mobile phones to improve young people’s sexual and reproductive health in low- and middle-income countries: a systematic review protocol to identify barriers, facilitators and reported interventions

**DOI:** 10.1186/s13643-019-1033-5

**Published:** 2019-05-18

**Authors:** Anam Feroz, Farina Abrejo, Sumera Aziz Ali, Rozina Nuruddin, Sarah Saleem

**Affiliations:** Department of Community Health Sciences, The Aga Khan University, Stadium Road, PO Box 3500, Karachi, 74800 Pakistan

**Keywords:** Mobile phones, mHealth, Young people sexual & reproductive health, Low- and middle-income countries, Systematic review, Facilitators, Barriers

## Abstract

**Background:**

Due to a growing reliance on mobile phone technology and decreasing mobile phone costs, the use of mobile phones is on the rise, especially among the youth population. Young people are responsive and enthusiastic to use novel approaches such as mHealth to access sexual and reproductive health information and services. Globally, reproductive health programs have used mHealth to provide sexual and reproductive health education and services to young people, through diverse communication channels. However, few attempts have been made to systematically review the mHealth programs for young people sexual and reproductive health (SRH) in low- and middle-income countries (LMICs). In addition, very little is known regarding the potential barriers and facilitators to the uptake of mobile phone interventions for improving young people SRH. This review aims to highlight facilitators and inhibitors to implementing and increasing uptake of mHealth interventions for young people’s SRH, in LMICs specifically. Additionally, the review will identify the range of mHealth solutions which can be used for improving young people’s SRH in LMICs.

**Methods:**

The review will focus on comparing the various types of mHealth interventions/strategies that are used to improve young people’s SRH services in LMICs. PubMed, CINAHL Plus, Science Direct, Cochrane, and gray literature will be explored using a detailed search strategy. The studies involving young people (adolescents and youth) aged 10–24 years to which mHealth interventions were delivered for improving their SRH outcomes will be included in this review. LMICs will be selected according to the World Bank’s (WB) 2018 Country Classification list. Studies published between January 2005 and March 2018 will be included as the field of mHealth has emerged over the last decade. English language articles will be included as the authors are proficient in this language.

**Discussion:**

The systematic review will assist researchers and SRH professionals in understanding facilitators and barriers to implementing and increasing the uptake of mHealth interventions for SRH in LMICs. Finally, this review will provide more detailed information about embracing the use of mobile phones at different levels of the healthcare system for improving young people’s SRH outcomes.

**Systematic review registration:**

PROSPERO CRD42018087585

**Electronic supplementary material:**

The online version of this article (10.1186/s13643-019-1033-5) contains supplementary material, which is available to authorized users.

## Background

Globally, a large number of young people are sexually active; the proportion of sexual activity differs substantially by region and gender [1]. More boys are sexually active than girls, in the Latin America and Caribbean region, where as girls are more sexually active in sub-Saharan African, Asian, and Central Asian regions [1]. Additionally, the proportion of sexual activity is increasing gradually from middle to late adolescent years [1, 2]. The early sexual debut results in unintended pregnancies and early childbearing and increases the risk of sexually transmitted infections (STIs) [3]. In developing countries, around 19% of girls become pregnant before age 18 and the adolescent birth rate is about 95% [3, 4]. In LMICs, most young people have very limited or no access to sexual and reproductive health (SRH) education and services, largely due to a lack of awareness, social stigma, policies and procedures inhibiting provision of contraception and abortion services to girls, and the judgmental attitudes of healthcare professionals [3, 5]. Thus, young people have special SRH education needs that remain unmet and to address these specific SRH needs, the use of innovative and novel approaches are required to ensure access to safe, effective, affordable, and acceptable SRH services [5].

Mobile health (mHealth) is defined as a “medical and public health practice supported by mobile devices, such as mobile phones, patient monitoring devices, personal digital assistants (PDAs), and other wireless devices” [6]. mHealth’s approach is increasingly being used in healthcare delivery. mHealth involves the use of mobile technologies and multimedia tools to support the accomplishment of health goals [7]. Many LMICs with restricted internet or print resources have attained a substantial level of cell phone penetration [8]. According to the International Telecommunication Union’s (ITU) 2016 report, globally, the total number of cell phone subscriptions has reached 5 billion people, and this number is likely to increase and surpass the world population in upcoming years due to growing reliance on mobile phone technology and decreasing mobile phone costs [9, 10]. In LMICs, cell phone penetration has surpassed over 90% in recent years [11]. On account of rapid expansion of mobile phone penetration and ownership in LMICs, the novel field of mHealth has gained much progress and it is being used rapidly in hundreds of diverse health-related projects [7].

The increased mobile phone penetration has led to a rise in mobile phone use, especially among the young population in LMICs [12, 13]. A survey was conducted in 24 developing nations to assess cell phone ownership. The survey report revealed that more than half of the population in each of the countries surveyed confirms that they own a cell phone. Moreover, a median of 78% of cell phone users across the 24 nations use short messaging service (SMS), making it the most popular communication method [14]. SMS is also reported to be the most common method of communication among African cell phone owners aged 18–34 years [15].

Young people are responsive and enthusiastic to use new innovative technologies such as mHealth to address barriers to receiving SRH information and services [16, 17]. The mHealth technology can help overcome most of the barriers including provider prejudice, stigmatization, discrimination, fear of refusal, lack of privacy and confidentiality, embarrassment in seeking SRH education and services on highly sensitive topics, cost prohibitions, and transportation challenges by providing safe, accurate, cost-effective, timely, and tailored young people’s SRH services [18]. More importantly, mHealth offers privacy, convenience, and easy access in contrast to face-face consultations with healthcare professionals, which eventually addresses the barriers of stigmatization and embarrassment in receiving tailored SRH services [19]. Worldwide, diverse mHealth solutions have been used to connect the young population to SRH information and services [20]. In LMICs, mHealth technology can be used to reach out to the youth population and to engage them to provide acceptable, safe, cost-effective, and accurate SRH services [18, 21].

The perceived benefits of mobile phone-based health interventions carry a great potential for improving young people’s SRH outcomes in LMICs. In an effort to tap into the potential of mHealth for young people SRH services, there has been an increase in the amount of research in recent years; while published studies from high-income countries (HIC) on mHealth interventions for young people SRH are growing, gaps in evidence exist related to mHealth for young people’s SRH in LMICs. In previous studies, attempts have been made to review the mHealth programs for young people’s SRH using the mHealthevidence.org website and through a global call for collecting information on mHealth interventions [22, 23]. A systematic review by L’Engle and colleagues assessed strategies on using mHealth to improve young people’s SRH by using the mHealth evidence reporting and assessment (mERA) checklist; although only three out of the 35 articles included in the review were related to LMICs, the small number of articles reflected the lack of literature from LMICs [22]. Another review by Ippoliti & L’Engle summarized 17 projects which involved mHealth interventions to improve young people’s SRH in LMICs, through the aforementioned global call for information. Both of these reviews included evidence regarding the use of mHealth for improving young people’s SRH. However, very little is known regarding the potential barriers and facilitators to the uptake of mobile phone interventions for improving young people’s SRH. This systematic review aims to highlight potential barriers and facilitators to the uptake of mHealth interventions for young people’s SRH, particularly in LMICs. Additionally, the review will compare the range of mHealth solutions which can be used for improving young people SRH.

## Methods

Labrique and colleagues identified 12 mHealth applications to respond to various health issues [24]. Few healthcare programs involve one application, while others may include two or more mHealth applications for addressing a particular health issue. The classification of 12 mHealth applications as per Labrique and colleagues is illustrated in Table 1. The same framework will be used to categorize the range of mHealth interventions which can be used to improve young people SRH.

**Table 1:** Eligibility criteria

Attribute	Inclusion criteria	Exclusion criteria
Population	Various terms are used to categorize young people: “adolescents” refers to 10–19 years, “youth” refers to 15–24 years, and “young people” refers to 10–24 years. Studies involving young people (adolescents and youth) aged 10–24 years to which mHealth interventions were delivered for improving their SRH outcomes	Studies involving groups of women, men, and girls under the age of 10 years and over the age of 24 years
Intervention	Studies will be included that have involved mHealth intervention to improve ASRH services	Studies involving other ICT interventions, ART compliance reminders, EmONC coverage, managerial and financial level interventions, physical mobile clinics, and teleconsultations
Comparison	The comparison is the usual standard of care, or in the case of a randomized control trial, the comparison is the control condition.	Not applicable
Outcome	Improvement in adolescent sexual and reproductive health services Behavioral outcomes Improved education and awareness ASR Health outcomes	Studies with other outcomes such as demonstrating skilled birth attendants, emergency care, quality of life, immunization coverage, cost-effectiveness of intervention, child development, and others
Setting	Studies conducted in LMICs	Studies conducted elsewhere
Study designs	Randomized and non-randomized controlled trials, pre- and post-test designs, non-experiment observational (cross-sectional, case-series, case studies), and qualitative papers	Commentaries, editorials, symposium proceedings, and systematic reviews
Language	Studies available in the English language as authors are proficient in this language	Studies which were not available in an English translation
Time period	Studies published between January 2005 to March 2018 as the field of mHealth emerged over the last decade	Studies published before January 2005 and after March 2018

**Fig. 1:**
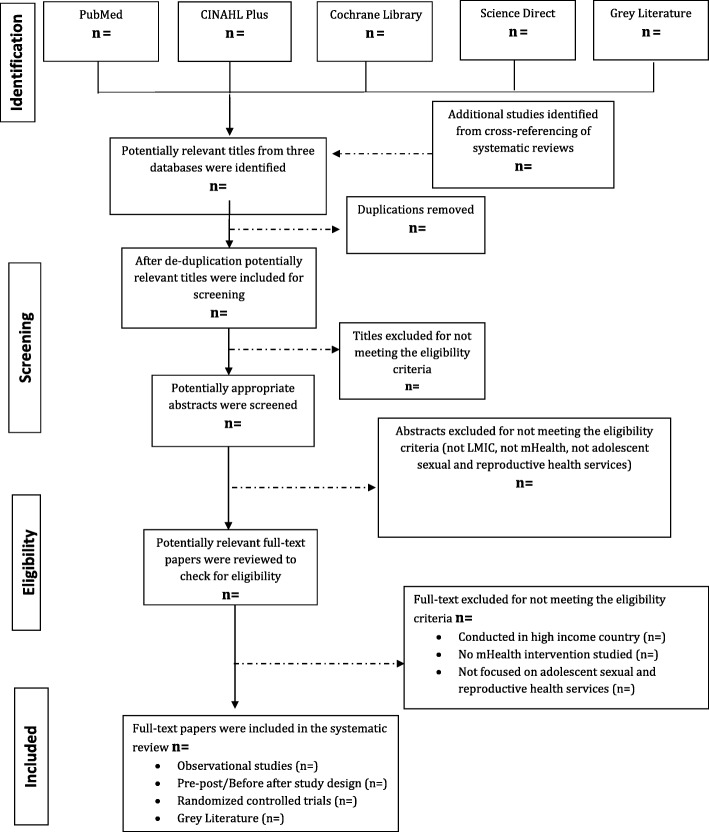
PRISMA flow diagram for database search of studies

The protocol has been designed and reported according to the Preferred Reporting Items for Systematic Reviews and Meta-analyses Protocols (PRISMA-P) checklist [25] (Fig. 1). The review protocol has been registered in the International Prospective Register for Systematic Reviews (PROSPERO) CRD42018087585 on Feb 5, 2018. The review will focus on examining the barriers and facilitators to implementation of mHealth programs for young people SRH in LMICs. Additionally, this review will help the research community in making decisions, regarding new methodologies and mobile phone interventions to be used to encourage the youth population to seek SRH information and services.

### Eligibility criteria

The studies involving young people (adolescents and youth) aged 10–24 years to which mHealth interventions were delivered for improving their SRH outcomes will be included in this review. LMICs will be selected according to the World Bank’s (WB) 2018 Country Classification list [26]. Issues concerning the use of mobile phones for young people SRH are common across many lower-middle-income economies [23]; thus these studies are more comparable than those led in HIC. Those studies will be included that have defined the use of mobile phone to improve young people’s SRH services and included behavior-, health-, and education and awareness-related outcomes from the mobile-based health interventions. Additionally, studies will be included that have identified common barriers and facilitators for implementation of mHealth interventions for young people’s SRH. For young people’s SRH outcomes, the review will use the United Nations Population Fund (UNPF) explanation which states that “Providing access to comprehensive sexuality education; services to prevent, diagnose and treat STIs; and counseling on family planning”. The UNPF also advocates that young people should be empowered so that they know their rights—including the right to delay marriage and the right to refuse unwanted sexual advances. Randomized controlled trials (RCTs), non-randomized studies, pre- and post-test designs, non-experiment observational (cross-sectional, case-series, case studies), and qualitative papers will be included in this review. Studies published between January 2005 and March 2018 will be included as the field of mHealth has emerged over the last decade. English language articles only will be included as the authors are proficient in this Language. Commentaries, editorials, symposium proceedings, and systematic reviews will be excluded in this review. The inclusion and exclusion criterion is illustrated in Table 1.

### Information sources and search strategy

An electronic systematic literature search will be carried out to explore the role of mobile health technology in improving young people SRH, particularly in LMICs. Although, there are a large number of databases on this pertinent topic; however, we will search four electronic databases including PubMed, CINAHL Plus, Science Direct, and Cochrane as they are generally considered large databases for systematic reviews. These databases will be explored using a detailed search strategy. Additionally, gray literature (non-published, internal, or non-reviewed papers, repositories) will also be explored as it is an important source for mHealth evaluations carried out in LMICs. The reference list of included records will also be appraised to identify relevant articles. Moreover, the reference lists of identified systematic reviews will also be reviewed to see if references include pertinent studies that might be included for review. The databases will be searched by two researchers independently. The search terms will be grouped under five major categories of interest: population (youth, adolescents, young people), intervention (mHealth), barriers and facilitators for implementation of mHealth interventions for SRH services, outcome (SRH), and settings (LMICs). Additionally, indexed keywords in the Medical Subject Headings (MeSH) will be used in order to ensure uniform search terms. The search strategy will be piloted to ensure sufficient specificity and sensitivity. The preliminary search strategy is illustrated in Table 2.

**Table 2:** Search strategy

Population	(‘adolescen*’ [Mesh] OR ‘school*age*’ OR student* OR teen* OR youth* OR ‘young adult*’ OR ‘young people’ OR ‘younger people’ OR ‘young women’ OR ‘young men’ ‘teenager’ OR ‘middle schooler’ OR ‘high schooler’ OR ‘secondary school’OR ‘Young adult’ [Mesh]) AND
Intervention	(Mobile phone OR mhealth [All Fields]) OR telemedicine [MeSH Terms]) OR cellphone [MeSH Terms]) OR reminder system [MeSH Terms]) OR wireless technology [MeSH Terms])OR text messaging [MeSH Terms]) OR medical informatics [MeSH Terms]) OR pda [MeSH Terms]) OR smartphone [MeSH Terms]) OR tablet computer [MeSH Terms]) AND
Barriers	Poor funding for SRH OR inadequate information on implementation costs OR inadequate information on cost-effectiveness OR political resistance OR poor availability of resources OR social unacceptability
Facilitators	Adequate information on implementation cost OR political stability and support OR availability of resources OR Social acceptability OR funding for SRH
Outcome	(Health outcomes OR behavioral outcomes OR Education and awareness OR ‘sexual health’ OR ‘reproductive health’ OR ‘sexual behavior’ OR ‘sex education’ OR condom* OR HIV OR HIV/AIDS OR PLHIV OR “acquired immunodeficiency syndrome” OR HPV OR ‘family planning’ OR abortion* OR abstinen* OR contracept* OR pregnan* OR sexual health rights OR ‘sexually transmitted infection’ OR ‘sexually transmitted infections’ OR STI OR STIs OR ‘sexually transmitted disease’ OR ‘sexually transmitted diseases’ OR ‘STD’ OR ‘STDs’ OR ‘sexual debut’ OR puberty OR ‘safe sex’) AND
Setting	(‘Developing country’ OR ‘South Asian countries’ OR ‘African countries’ OR ‘low and middle income Arab Countries’ OR ‘developing nation’ OR ‘least developed country’ OR ‘least developed nation’ OR ‘less developed nation’ OR ‘third world country’ OR ‘third world nation’ OR ‘under developed country’ OR ‘remote region’ OR ‘low and middle income country’ OR ‘under developed nation’ OR ‘low and middle income nation’ OR Angola OR Indonesia OR Philippines OR Armenia OR Jordan OR São Tomé and Principe OR Bangladesh OR Kenya OR Solomon Islands OR Bhutan OR Kiribati OR Sri Lanka OR Bolivia Kosovo OR Sudan OR Cabo Verde OR Kyrgyz Republic OR Swaziland OR Cambodia OR Lao PDR OR Syrian Arab Republic OR Cameroon OR Lesotho OR Tajikistan OR Congo, Rep. OR Mauritania OR Timor-Leste OR Côte d’Ivoire OR Micronesia, Fed. Sts. Tunisia OR Djibouti OR Moldova OR Ukraine OR Egypt, Arab Rep. OR Mongolia OR Uzbekistan OR El Salvador OR Morocco OR Vanuatu OR Georgia OR Myanmar OR Vietnam OR Ghana OR Nicaragua OR West Bank and Gaza OR Guatemala OR Nigeria OR Yemen, Rep. OR Honduras OR Pakistan OR Zambia OR India OR Papua New Guinea)
Filters	Publication date from January 2005 to March, 2018; Humans; English

### Study selection

Citation management system (Endnote software) will be used to manage the records exported from all the electronic databases [27]. In the first step, all the studies will be screened by study titles using the Endnote software. The shortlisted studies will then be screened by study abstracts. Lastly, the full text of selected studies will be retrieved and screened against the eligibility criteria. In order to ensure the reliability of screening articles among the two reviewers, a pre-defined screening form will be developed and pilot testing will be conducted as per the eligibility criteria. Both reviewers will describe outcome measures after reviewing the studies to verify the relevance of the articles. Strong justifications for excluding studies will be provided by each reviewer. Any disagreement between the two reviewers will be resolved by a third reviewer in a consensus meeting. The third reviewer will be consulted to make the final decision about whether the study meets the eligibility criteria for inclusion. The PRISMA flow diagram will be used to report the study selection process.

### Data collection process

A customized data extraction sheet will be filled by two independent reviewers (AF, FA) for the eligible studies. Data extraction tables of both reviewers will be matched to ensure that all key findings are included in the systematic review. A third evaluator will be involved, if discordant information is observed during the data extraction process. The preliminary data extraction table is illustrated in Additional file 1: Table S1. The data extraction sheet will be pilot tested before initiating the data extraction process. Alongside, existing studies on this research area have been reviewed to determine items of the data extraction form. The items included in the preliminary data extraction form include the title of the article, author, publication date, country of study, date of extraction, reviewer name, purpose/aim of the study, study type, study population, type of mHealth intervention used, study outcomes—improvement in young people’s SRH services, study limitations, included/excluded, reason for exclusion, and quality appraisal of included studies. The summary of included studies on mHealth interventions to improve young people’s SRH will also be provided in the main results paper.

### Quality assessment of included studies

To assess the methodological quality of the included studies, standardized quality assessment tools will be utilized. To evaluate the risk of bias in RCTs, The Cochrane Collaboration’s tool will be used [28]. This tool helps assess seven specific domains, namely sequence generation, allocation concealment, blinding of participants and personnel, blinding of outcome assessment, incomplete outcome data, selective outcome reporting, and other potential threats to the study’s validity. The tool is divided in to two parts. The first part of the tool outlines what was reported in the study in sufficient detail to support a judgment regarding the risk of bias. The second part of the tool gives a judgment relating to the risk of bias. This is achieved by assigning a judgment of “low risk,” “high risk,” or “unclear risk.” For this tool, a low risk of bias is the best possible rank representing a higher quality of study. The methodological quality of non-randomized studies will be examined through the ROBINS-I tool [29]. This tool is used to evaluate various aspects of methodological quality such as participant selection, measurement of intervention, variations from intended interventions, missing data, measurement in outcomes, and selection of the reported result. Each study will be rated as critical, serious, moderate, or low risk of bias based on a judgment of the collected information. If there are inadequate details, the risk of bias will be classified as “no information” or the corresponding study authors will be contacted for further information. Two reviewers (AF, FA) will independently assess the risk of bias of the included studies. If a disagreement occurs between the two reviewers, a third reviewer will be consulted (RN). Data on the risk of bias will be provided in an additional table for all the included studies.

### Synthesis of included studies

First, the findings of the review will be synthesized narratively. Initially, we will perform a descriptive analysis of all the final included studies to record their main characteristics such as study title, authors, publication year, study aim, study methods, sampling strategy, characteristics of study participants, types of SRH interventions, and study outcomes. Then, narrative synthesis will be carried in which final studies will be grouped under main mHealth applications, as defined in the Labrique and colleagues framework. The framework will be adapted based on the emerging themes. This process is usually used to identify main themes from qualitative and quantitative studies. Firstly, the two independent reviewers (AF, FA) will read each included study several times to extract data and group-related results. Later, the reviewers will record analytical interpretations of findings to capture emerging themes. Finally, the reviewers will highlight potential facilitators and inhibitors to the uptake of mHealth interventions for young people SRH in LMICs [30].

For the quantitative studies, we will conduct a sub-group analysis under different categories including barriers, facilitators and interventions. Under these categories, measures of associations such as odds ratios, relative risks, and prevalence ratios will be synthesized and reported for associations between barriers, facilitators, and interventions with proposed study outcome of sexual and reproductive health of youngsters. Moreover, we will also provide a narrative of confounders or effect modifiers being adjusted in different quantitative studies to highlight the importance of independent barriers, facilitators, and interventions important to improve the sexual and reproductive health of young people.

## Discussion

A more comprehensive understanding of the role of mobile phones for improving young people SRH is required, especially in LMICs. The protocol will lead to a systematic review which synthesizes evidence on the types of mHealth interventions used at different levels of the health care system to provide SRH education and services to young people in urban and rural communities of LMICs. The review will increase our understanding on how mHealth interventions targeted to the youth population help overcome barriers of provider prejudice, stigmatization, discrimination, fear of refusal, lack of privacy and confidentiality, cost prohibitions, and transportation challenges. Systematic review findings will be made publicly available. The results of the review will be disseminated through presentations and peer-reviewed publications.

## Additional file

Additional file 1: **Table S1.** Data extraction form (DOCX 24 kb)

## Abbreviations

HIC: High-income countries
LMICs: Low- and middle-income countries
PRISMA-P: Preferred Reporting Items for Systematic Reviews and Meta-analyses Protocols
RCTs: Randomized controlled trials
SRH: Sexual and reproductive health
UNPF: United Nations Population Fund
WB: World Bank
