# Supplementary material for: Using mobile phones to improve young people’s sexual and reproductive health in low- and middle-income countries: a systematic review protocol to identify barriers, facilitators and reported interventions
Source: Syst Rev. 2019 May 18;8:117. doi: 10.1186/s13643-019-1033-5 (PMC6525977; doi:10.1186/s13643-019-1033-5)
Supplement: Supplementary file 1 — Table S1. Data extraction form (DOCX 24 kb) [file 13643_2019_1033_MOESM1_ESM.docx]

**Table S3 Data Extraction Form**

| Study ID: | | Date form completed: |
| --- | --- | --- |
| First author: | Year of study: | Data extractor: |
| Citation: | | |

1. General Information

| Publication type Journal Article ⬜ Abstract ⬜ Other (specify e.g. book chapter)___________________ | |
| --- | --- |
| Country of study: | |
| Funding source of study: | Potential conflict of interest from funding? Y / N / unclear |

2. Study Eligibility

| Study Characteristics | | | | Page/ Para/ Figure # |
| --- | --- | --- | --- | --- |
| Type of study  (Original studies, case studies, cross-sectional studies, case-control studies, randomized controlled trials, quasi experimental studies, before and after (pre-post studies), qualitative formative studies, and clinical control trials) | ⬜ Randomised Controlled Trial (RCT)  ⬜ Cluster Randomised Controlled Trial (cluster RCT) | | ⬜ Controlled Before and After (CBA) study   - Contemporaneous data collection - Comparable control site - At least 2 x intervention and 2 x control clusters |  |
|  | ⬜ Qualitative study   - Exploratory - Descriptive - Ethnography - other | | ⬜ Other design (specify): |  |
|  | *Does the study design meet the criteria for inclusion?*  Yes ⬜ No ⬜ 🡪Exclude Unclear ⬜ | | |  |
| Participants  (Studies involving young people (adolescents and youth) ages between 10–25 years to which m-Health interventions were delivered for improving their SRH outcomes) | Describe the participants included: | | |  |
|  | Are participants defined as a group having specific demographic, social or cultural characteristics? | Yes ⬜ No ⬜ Unclear ⬜  Details: | |  |
|  | How is the geographic boundary defined? | Details:  Specific location (e.g. state / country): | |  |
|  | *Do the participants meet the criteria for inclusion?* | Yes ⬜ No ⬜ 🡪Exclude Unclear ⬜ | |  |
| Types of intervention  (Studies will be included that has involved mHealth intervention to improve ASRH services) | Strategies included in the intervention |  | |  |
|  | Focus of the intervention |  | |  |
|  | *Does the intervention meet the criteria for inclusion?* | Yes ⬜ No ⬜ 🡪Exclude Unclear ⬜ | |  |
| Types of outcome measures  (Outcomes demonstrating improvement in adolescent sexual and reproductive health services such as the right to delay marriage, the right to refuse unwanted sexual advances, access to comprehensive sexuality education, services to prevent, diagnose and treat STIs, and counseling on family planning) | List outcomes: |  | |  |
|  | Outcome measured at a population level or individual level? | Details: | |  |
|  | *Do the outcome measures meet the criteria for inclusion?* | Yes ⬜ No ⬜ 🡪Exclude Unclear ⬜ | |  |

Summary of Assessment for Inclusion

| Include in review ⬜ Exclude from review ⬜ | |
| --- | --- |
| Independently assessed, and then compared?  Yes ⬜ No ⬜ | Differences resolved Yes ⬜ No ⬜ |
| Notes: | |

DO NOT PROCEED IF PAPER EXCLUDED FROM REVIEW

## 3. Study Details

|  | Descriptions as stated in report/paper | | Location in text or source *(pg & /fig/table/other)* |
| --- | --- | --- | --- |
| Aim of study |  | |  |
| Study Design |  | |  |
| Data collection methods |  | |  |
| Study Duration |  | |  |
| Start date |  | |  |
| End date |  | |  |
| Ethical approval needed/ obtained for study | Yes No Unclear |  |  |
| Notes: | | | |

## Participants

|  | Description  *Include comparative information for each intervention or comparison group if available* | | Location in text or source *(pg & /fig/table/other)* |
| --- | --- | --- | --- |
| Population description |  | |  |
| Setting |  | |  |
| Inclusion criteria |  | |  |
| Exclusion criteria |  | |  |
| Method of recruitment of participants |  | |  |
| Informed consent obtained | Yes No Unclear |  |  |
| Total no. randomised  *(or total pop. at start of study for NRCTs)* |  | |  |
| Clusters  *(if applicable, no., type, no. people per cluster)* |  | |  |
| Withdrawals and exclusions |  | |  |
| Age |  | |  |
| Sex |  | |  |
| Race/Ethnicity |  | |  |
| Severity of illness |  | |  |
| Co-morbidities |  | |  |
| Other relevant sociodemographics |  | |  |
| Notes: | | | |

## Intervention groups

*Copy and paste table for each intervention and comparison group*

Intervention Group 1

|  | Description as stated in report/paper | Location in text or source *(pg & /fig/table/other)* |
| --- | --- | --- |
| Group name |  |  |
| No. randomised to group  *(specify whether no. people or clusters)* |  |  |
| Description *(include sufficient detail for replication)* |  |  |
| Duration of intervention period |  |  |
| Timing *(e.g. frequency, duration of each episode)* |  |  |
| Delivery *(e.g. mechanism, medium, intensity, fidelity)* |  |  |
| Providers  *(e.g. no., profession, training, ethnicity etc. if relevant)* |  |  |
| Co-interventions |  |  |
| Integrity of delivery |  |  |
| Compliance |  |  |
| Notes: | | |

## Outcomes

Outcome 1

|  | Description as stated in report/paper | | Location in text or source *(pg & /fig/table/other)* |
| --- | --- | --- | --- |
| Outcome name |  | |  |
| Outcome definition |  | |  |
| Person measuring/ reporting |  | |  |
| Unit of measurement  *(if relevant)* |  | |  |
| Is outcome/tool validated? | Yes No Unclear |  |  |
| Notes: | | | |

## For qualitative studies

| Which key themes are stated to have emerged from the qualitative research? |  |
| --- | --- |
| Facilitators to implementing and increasing uptake of mHealth interventions for ASRH, particularly in LMICs. |  |
| Barriers to implementing and increasing uptake of mHealth interventions for ASRH, particularly in LMICs. |  |

## Other

| Study funding sources  *(including role of funders)* |  |  |
| --- | --- | --- |
| Possible conflicts of interest  *(for study authors)* |  |  |
| Notes: | | |
